# Dysfunction of the Blood-brain Barrier in Cerebral Microbleeds: from Bedside to Bench

**DOI:** 10.14336/AD.2021.0514

**Published:** 2021-12-01

**Authors:** Hai-ling Wang, Chun-lin Zhang, Yan-mei Qiu, An-qi Chen, Ya-nan Li, Bo Hu

**Affiliations:** Department of Neurology, Union Hospital, Tongji Medical College, Huazhong University of Science and Technology, Wuhan 430022, China.

**Keywords:** Blood-brain barrier, cerebral microbleeds, cerebral amyloid angiopathy, hypertensive vasculopathy, endothelial dysfunction

## Abstract

Cerebral microbleeds (CMBs) are a disorder of cerebral microvessels that are characterized as small (<10 mm), hypointense, round or ovoid lesions seen on T2*-weighted gradient echo MRI. There is a high prevalence of CMBs in community-dwelling healthy older people. An increasing number of studies have demonstrated the significance of CMBs in stroke, dementia, Parkinson’s disease, gait disturbances and late-life depression. Blood-brain barrier (BBB) dysfunction is considered to be the event that initializes CMBs development. However, the pathogenesis of CMBs has not yet been clearly elucidated. In this review, we introduce the pathogenesis of CMBs, hypertensive vasculopathy and cerebral amyloid angiopathy, and review recent research that has advanced our understanding of the mechanisms underlying BBB dysfunction and CMBs presence. CMBs-associated risk factors can exacerbate BBB breakdown through the vulnerability of BBB anatomical and functional changes. Finally, we discuss potential pharmacological approaches to target the BBB as therapy for CMBs.

## 1. Introduction

**Figure 1.**
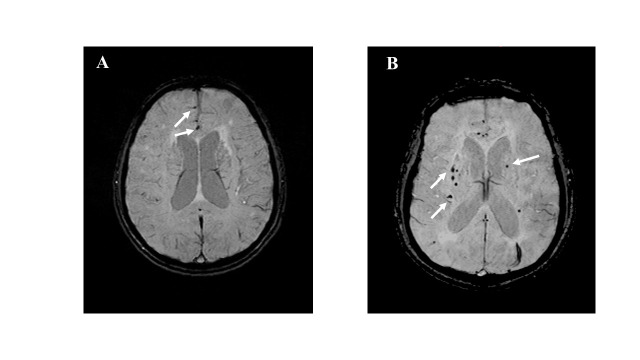
**Representative images of cerebral microbleeds (CMBs) visualized on susceptibility-weighted images (SWI).** (white arrow). **(A)** lobar microbleeds, **(B)** deep cerebral microbleeds.

Cerebral microbleeds (CMBs) are designated as small (<10 mm), hypointense, round or ovoid lesions detectable by T2*-weighted gradient echo MRI, which have been increasingly detected with the widespread application of high blood-sensitive MRI techniques, such as T2*-weighted gradient-recalled echo (GRE) and susceptibility-weighted imaging (SWI) (Fig. 1) [1, 2]. Histopathological investigations have demonstrated that CMBs, which are punctuate hemorrhagic lesions, contain hemosiderin deposits most likely resulting from the leakage of erythrocytes from small cerebral vessels, such as arterioles and capillaries [3, 4]. The pathophysiology of CMBs is varied with their location. The lobar microbleeds are related to cerebral amyloid angiopathy (CAA), while deep or mixed CMBs are attributable to hypertensive vasculopathy [2, 5]. Because the presence of CMBs is regarded as a precursor of both intracerebral hemorrhage (ICH) and ischemic stroke, there has been increased research on CMBs [5-7]. There is an increased prevalence of CMBs in patients with stroke. The frequency of CMBs is as high as 50% -80% in patients with primary ICH and approximately 35%~71% in patients with ischemic stroke [8]. The presence of CMBs, especially in a population with higher CMBs counts, is associated with increased risks of all subtypes of stroke, and this depends on whether or not the location is typically affected by CAA [5]. More remarkably, in adults with recent ischemic stroke or transient ischemic attack (TIA), the CMBs burden corresponds to a more significant relative hazard for subsequent ICH than for ischemic stroke, but a higher absolute risk of ischemic stroke than that of ICH regardless of the count and anatomical patterns of CMBs [6]. Meanwhile, CMBs can also contribute to neurologic dysfunction, and previous studies have found that increased CMBs burden is associated with cognitive deterioration and dementia [9-12]. Therefore, the pathogenesis of CMBs involves damage to the vascular wall as a result of both vascular risk factors and accumulation of β-amyloid (Aβ), and the presence of CMBs is regarded as a marker of diffuse vascular and neurodegenerative brain damage [9, 13].

The risk factors contributing to microbleeds include age, blood pressure, diabetes mellitus, low serum cholesterol, smoking, and apolipoprotein E (APOE) genotype [13-16]. Among all of the above, cardiovascular risk factors contribute to deep or infratentorial microbleeds, while APOE genotype is associated with strictly lobar CMBs [15]. In addition, aging is commonly accepted as an independent risk factor related to CMBs.

At all cerebrovascular tree levels, the blood-brain barrier (BBB) is characterized as a dynamic and metabolic interface between the blood and the central nervous system (CNS). The BBB comprises a single layer of brain endothelial cells (ECs) lining up to form the cerebral blood vessels, with close communication with astrocytes, pericytes, and the basement membrane. The pericytes are embedded in the basement membrane of brain ECs, while the astrocytic endfeet almost wholly envelop the capillaries as the outer surface of the BBB [17-20]. The BBB plays an essential role in maintaining homeostasis in the CNS by preventing neurotoxic components in the blood, blood cells, and pathogens from entering the brain, a prerequisite of normal neurological function [21-23]. BBB dysfunction, with the loss of physiological functions and structural integrity, is a significant pathological characteristic of many cerebral disorders. Multiple studies have also demonstrated the existence of BBB leakage in CMBs.

Despite recognition from an increasing number of researchers regarding the crucial clinical significance of CMBs, there currently are no proper animal models for CMBs, and the etiological mechanism behind CMBs has not been elucidated clearly. In this review, we provide clinical knowledge and theories regarding CMBs, including their clinical epidemiology, risk factors, and pathogenesis. We also summarize how CMBs-related risk factors contribute to BBB dysfunction and provide additional detail regarding CMBs-related cerebrovascular dysfunction, focusing on BBB permeability, endothelial dysfunction, and molecular and cellular mechanisms of vascular disease.

## 2. Introduction to cerebral microbleeds

### 2.1 The pathogenesis of cerebral microbleeds

Cerebral microbleeds are defined as small (<10 mm), hypointense (black seen on T2*-weighted MRI), round or ovoid pathological lesions with associated blooming detectable by T2*-weighted MRI techniques, but T1- or T2-weighted sequences [2, 24]. However, the term microbleeds was first denoted as homogeneous, small (2 to 5 mm in diameter seen on imaging), hypointense, round lesions on T2-weighted imaging by Offenbacher et al. in 1996 [25]. With the advances in MRI techniques, especially GRE and SWI with high sensitivity to the hemosiderin deposits, the detection of CMBs increases rapidly, facilitating the evolution of CMBs identification criteria [2].

However, the pathogenesis of CMBs has not yet been elucidated clearly. Histopathological studies have shown that CMBs contain hemosiderin deposits or hemosiderin-laden macrophagocytes adjacent to abnormal small cerebral vessels presenting with fibrolipohyalinosis or cerebral amyloid angiopathy. CMBs appear to be a marker of increased vascular fragility [3, 4]. Therefore, cerebral microbleeds seem to indicate the occurrence of previous extravasation of erythrocytes from small cerebral vessels, as a consequence of bleeding-prone cerebral arteriopathies, including hypertensive vasculopathy and cerebral amyloid angiopathy [1, 3, 4]. Contrary to ICH, the mechanical injury to brain tissue caused by microbleeds is barely measurable, due to their limited hematoma. However, products derived from blood extravasations, especially ion, can lead to a series of secondary brain injuries, such as BBB breakdown and inflammatory activation [26]. The pathophysiology of CMBs might last for an extended period, including dendritic degeneration, microglial activation, and iron deposit. Irons accumulation after multiple micro-hemorrhages leads to a series of secondary brain damage and impairs spatial cognition [27]. In addition, the location of CMBs corresponds to two different types of underlying vasculopathy. Strictly lobar CMBs, regardless of cerebral or cerebellar compartments, appear to result from CAA, referring to the damage of cortical and leptomeningeal vessels due to the increasing amyloid load. Population-based studies have investigated the association between CMBs and CAA locations, where the presence of new lobar CMBs was consistent with higher baseline β-amyloid load [28-30]. The presence of CMBs in deep or infratentorial areas (with or without lobar CMBs) is attributed to hypertensive vasculopathy and corresponds to vascular lesions within superficial perforating arterioles [31, 32]. Besides, as a bleeding-prone vasculopathy, microbleeds are regarded as a subclinical precursor of ischemic or hemorrhagic stroke. The strictly lobar CMBs, which are always present with CAA, are suggested to increase ICH risk. Simultaneously, cerebral microhemorrhage located in other regions is related to both ischemic and hemorrhagic stroke [5].

#### 2.1.1 Cerebral amyloid angiopathy

Cerebral amyloid angiopathy is characterized by a cerebrovascular disease with the accumulation of aggregated β-amyloid protein, which selectively involves the parenchymal and leptomeningeal vessel walls. The establishment of the Boston criteria makes it possible to understand more about the pathological mechanisms of CAA [33]. Recently, a study based on a transgenic rat model of CAA (rTg-DI) showed cerebral microbleeds were present about three months after reduced cerebrospinal fluid (CSF) /plasma Aβ40 levels [34]. The primary form of vascular Aβ deposition in CAA is composed of Aβ40, which is the product of amyloid precursor protein (APP) cleaved by β-secretase 1 (BACE-1) and γ-secretases [35, 36]. Soluble Aβ is eliminated by enzymatic breakdown, BBB clearance, interstitial fluid bulk-flow clearance, perivascular drainage, phagocytosis, and CSF absorption [37]. Among all of the reported clearance pathways, perivascular drainage pathways, driven by pulsations of the blood vessel wall, are believed to contribute primarily to the pathogenesis of CAA [38, 39]. Moreover, extracellular iron dyshomeostasis might contribute to excessive amyloid plaques in CMBs. This process can be explained by decreasing iron efflux mediated by the APP/Fpn1 complex and increased affinity of APP/BACE1 [40]. Additionally, Aβ deposition predominantly affects arterioles, as the major pathways for perivascular Aβ clearance [41]. The Aβ peptides are able to spread along perivascular drainage pathways to accumulate in the walls of vessels and form a self-reinforcing cycle. The vascular deposition of Aβ generates the loss of smooth vascular cells, leading to a further reduction in Aβ elimination [38, 42]. The Aβ deposition in the basement membranes of parenchymal and leptomeningeal vessels progressively replaces the smooth muscle cells located in the tunica media, until it finally composes the entire vessel wall [43]. Consequently, Aβ deposition leads to the rupture of small vessels, the accumulation of amyloid peptides in the brain, and inflammatory processes [44-48]. Moreover, these changes are suggested to influence the integrity of BBB, leading to the extravasation of proteinaceous fluid and blood cells. BBB disruption might be a contributory mechanism for CAA-related brain injury [38]. Furthermore, Aβ deposition in CAA usually affects the cortico-subcortical brain regions rather than the deep or infratentorial areas [49, 50].

Endothelial dysfunction is one of the most important characteristic features in the pathogenesis of CAA. On the one hand, Aβ deposition-induced BBB hyperpermeability may partly be attributed to endothelial death [51]. Aβ deposition may disrupt endothelial mitochondrial metabolic pathways by inhibiting the conserved metabolic enzyme activity involved in the tricarboxylic acid cycle, electron transport chain, and oxidative phosphorylation in a manner similar to that in the mitochondria of other cell types [52, 53]. On the other hand, Aβ deposition-related BBB dysfunction is attributed to increased pinocytotic vesicles and loss of tight junction (TJ) proteins [54, 55]. Aβ upregulates the endothelial expression of matrix metalloproteinase (MMP)-2 and MMP-9 through binding to RAGE, which is involved in the degradation of TJ proteins and vascular inflammation [56, 57]. Aβ deposition promotes the expression of MCP-1, GRO, IL-1β, and IL-6 in brain ECs through the JNK-AP1 signaling pathway, recruiting peripheral immune cells into the cerebral parenchyma [58]. Retraction and swelling of astrocyte endfeet were observed prior to widely spread β-amyloid plaque pathology [59]. Aβ exposure induces the progressive loss of mitochondrial membrane potential and triggers the cell death pathway in astrocytes. Loss of astrocytic endfeet and loss of Aquaporin 4, Kir4.1, and dystrophin 1 localized to the astrocytic endfeet are observed in transgenic mouse models with amyloid deposition and postmortem brain tissues from AD patients [60]. Astrocytes increase the expression and secretion of MMP-2 and MMP-9 in CAA, which degrade both Aβ and TJ proteins [61]. In CAA, reactive astrocytes also activate transcription factor nuclear factor-kappa B (NF-κB), which is followed by elevated expression of TNF-α, IL-1β, and many other pro-inflammatory factors [62-65]. Aβ stimulates the release of endothelin-1 from pericytes, which leads to contraction of pericytes and capillaries [66]. Pericyte loss and insufficient vascular platelet-derived growth factor receptor-β (PDGFRβ) signaling is a typical change that occurs during the pathogenesis of CAA [67]. Pericytes may be dead from Aβ-mediated oxidative stress, excitotoxicity, and mitochondrial dysfunction in CAA [68, 69]. The reduced number and coverage of pericytes accelerate CAA due to their active transportation of Aβ across the BBB from the brain to the blood [70, 71].

CAA is a common age-related pathology. A community-dwelling study with a well-characterized older population showed that CAA pathology was prevalent with a frequency of 85% in people investigated, and present in 94% of those with dementia and 77% of those without cognitive disorders [72]. The CAA pathology in the elderly might be related to the reduction of Aβ clearance, and may be aggravated by aging, as a result of the thickening of vessel walls, loss of vasoactivity, and alteration of basement membrane proteins [38, 73-75]. In addition, numerous studies have shown the presence of CAA is associated with spontaneous lobar ICH, Alzheimer’s disease, and cognitive impairment [76-79]. Furthermore, CAA-related brain injury is also frequently present in small vessels diseases, such as lacunar infarcts, CMBs, white matter hyperintensity (WMH) [2, 43, 80, 81]. Aβ burden is associated with increasing frequency of CMBs, with a posterior cortical predominance [2, 28]. Studies in vivo about the association between CMBs and CAA have found that Aβ deposition in vessels is not sufﬁcient to account for vessels bleeding directly, while flow network dynamics may either make a contribution. CMBs are more likely to be present on bends and bifurcations, which are the anatomically vulnerable segments, as a consequence of continuous turbulence [41].

#### 2.1.2 Hypertensive vasculopathy

Contrary to lobar microbleeds, deep or infratentorial CMBs, present in the cerebellum, basal ganglia, thalamus, and brainstem, are associated with hypertensive vasculopathy [2]. Hypertensive vasculopathy, also known as hypertensive small vessel disease or arteriolosclerosis, negatively influences the blood supply of deep perforators [82]. The pathological changes of hypertensive vasculopathy are comprised of fibrinoid necrosis, lipohyalinosis, microatheroma, and microaneurysms [83]. Among all of the mechanisms mentioned above, fibrinoid necrosis, characterized by the accumulation of plasma proteins in vessel walls, causes the degeneration of the muscle and collagen to produce hyalinization. Additionally, autopsy pathology evidence suggests that in most lipohyalinosis, which is the deposition of fibro-hyaline materials in small perforating arteries, concentrical hypertrophy of the vessel wall and reduction of the inner arteriolar diameter occur, as a result of the loss of vascular smooth cells [84, 85]. Moreover, the narrowing of lumens plays a significant role in elevated cerebrovascular resistance, reduced autoregulatory capacity, and increased BBB leakage. In the development of hypertensive vasculopathy, the function of the microvascular endothelium decreases at the early stage due to the structural remodeling of cerebral small vessels via increased angiotensin II (Ang II). Moreover, the alteration of the basement membrane, the disruption of BBB, and the loss of autoregulation also contribute to the progress of hypertensive vasculopathy, with the entire microvessel wall damage followed [86, 87].

Dysfunction of BBB components, including endothelial cells, pericytes, and astrocytes, is well described in multiple hypertension models. Hypertension impairs the survival status, cerebral vascular blood regulating function, and barrier function of endothelial cells. First, hypertension exacerbates oxidative stress injury in endothelial cells through inhibition of the enzyme activity of superoxide dismutase and catalase, accompanied by decreased glutathione content and increased malondialdehyde level [88-90]. Transcriptomic profile analysis of cerebral endothelial cells indicates that hypertension activates pathways related to apoptosis and mitochondrial responses [91]. Second, hypertension impairs the expression of brain-derived neurotrophic factor (BDNF) and endothelial nitric oxide synthase (eNOS) in endothelial cells, which may account for the impaired dilatory capability of endothelial cells [92]. Third, endothelial paracellular and transcellular permeability are significantly impaired in hypertension, which is related to the decreased level of TJ proteins and elevated cerebral EC endocytosis [93, 94]. Pericytes degeneration and detachment are observed during the pathogenesis of hypertension [95]. The coverage rate of pericytes is much higher in the brain of spontaneously hypertensive rats (SHR) [96]. Pericytes overlay and encircle the endothelial cells more tightly and closely under hypertensive conditions [97]. Transcriptomic profile analysis reveals that hypertension causes upregulation of cell division signaling pathways and downregulation of cell adhesion signaling pathways in brain microvascular pericytes [98]. Swelling and pathological detachment of astrocytes endfeet were detected in hypertensive rats [95]. The astrocyte expression of AQP4 is upregulated in hypertension, which may accelerate brain edema [99]. Ang II-induced chronic hypertension mediated enhanced spontaneous Ca^2+^ events and augmented transient potential receptor vanilloid 4 channel expression in endfeet during parenchymal arteriole myogenic responses [100]. Hypertension also induces astrocyte activation and neuroinflammation in an Ang II-dependent manner [101].

Hypertension is regarded as a significant risk factor for arteriolosclerosis present in the brain and other organs, such as the kidney and retina [43]. Hypertension is the second largest risk factor of microbleeds after age. A study involving a hypertensive population without cerebrovascular disease history showed that the prevalence of CMBs was 16.1%, which was more than triples than that reported in the general population [102]. Furthermore, the presence of cerebral microbleeds plays a significant role in subsequent macrobleed and hemorrhage recurrence [2, 103]. It is reasonable to presume the similarity in the effects of hypertension on cerebral hemorrhage and microbleeds.

### 2.2 The animal models of CMBs

Due to the clinical importance of CMBs, it is urgent to find a stable and exact model to study the underlying mechanisms and related therapeutic interventions of CMBs in depth. There are several relevant experimental animal models designed in rodents from different procedures to mimic the performance of CMBs (Table 1).

First, Fisher et al. observed the progressive accumulation of microbleeds with aging in Tg 2576 transgenic mice, which are characterized by amyloid deposits in their leptomeningeal and cortical artery arterioles. In that study, 24-month old animals were more than twice as likely to develop CMBs of larger size as compared to younger adult mice [105]. Since then, more aged CAA-related animal models have been applied to study the temporal and spatial development of CMBs, including APPswe/PSEN1dE9 (APP/PS1) mice and APP23-transgenic mice [41, 105, 106]. Additionally, Rosidi et al. used femtosecond laser pulses to trigger cortical microhemorrhages that occurred because of the rupture of targeted small arterioles or capillaries. The focused laser pulses specifically targeted a single cortical penetrating arteriole to produce 100 µm diameter hematoma with specific spatial and temporal distribution and minimal harm to the surrounding tissues. Combined with applying two-photon excited fluorescence microscopy, researchers can track the physiological changes after microhemorrhages, such as bleeding dynamics, tissue compression, blood flow changes, and the dynamics of multiple cells in the brain [109]. In addition to CAA-related animal models, hypertension-induced cerebral microhemorrhages are also present in aged mice. The hypertensive mice model induced by Ang II and L-NAME (inhibitor of nitric oxide synthase) was initially used by the Heistad laboratory to mimic spontaneous intracerebral hemorrhage [110]. Toth et al. confirmed that all hypertension-induced spontaneous intracerebral hemorrhage mice developed multiple histologically detectable CMBs, and the number of CMBs tended to increase with aging [111]. Furthermore, Tarantini et al. observed similar phenomena in hypertensive mice with specific knockdown of insulin-like growth factor 1 (IGF-1), an important anabolic hormone that decreases with aging. IGF-1 deficiency can mimic the aging phenotype and increase the incidence of CMBs [112]. Regardless, the CAA- and hypertension-induced CMBs models mimick two types of CMBs pathological characteristics and take approximately 15 to 24 months are required for CMBs development. Therefore, researchers tried to develop new CMBs models that are easier to establish and require less time.

Hoffmann et al. established hypoxia-reoxygenation-induced microhemorrhage models in the process of studying the pathophysiology of high-altitude hypoxic brain injury. All adult mice were exposed to normobaric hypoxia at 8% oxygen for 48 hours and then were maintained for a further 24h at room air by rapid reoxygenation. Both image and histological analyses found the presence of CMBs after hypoxic exposure, and their number and size significantly increased after 24 hours of reoxygenation, especially in the olfactory bulb [111]. Sumbria et al. reported an inflammation-induced mouse model of CMBs. The mice were treated with intraperitoneal lipopolysaccharide (LPS) to mimic acute and sub-acute CMBs development by adjusting the dose regimen. LPS-induced CMBs are associated with endothelial activation and BBB damage. Compared to other existing models for CMBs, the LPS-induced mouse model has its unique advantages, including simplicity, feasibility, non-invasiveness, high success rate, and low mortality [112]. Recently, Bergeron et al. developed a reproducible murine model of collagenase-induced cortical CMBs by stereotaxic cortical injection of 0.8?µU collagenases. This new CMBs model is sensitive to pharmacological modulation and presents with cognitive impairments and hypometabolism six weeks after surgery. Therefore, this model might contribute to the progress of CMB treatment strategies, especially in the fields of vascular cognitive impairment [113].

Nevertheless, none of the existing animal models mentioned above can mimic spontaneous generation of CMBs without intervention and also cover all types of clinical CMBs. Additionally, some of them might be confounded by intracerebral macrohemorrhage. Currently, there is a lack of appropriate animal models to study how CMBs contribute to cognitive disorder and cerebrovascular disease, especially ICH. Therefore, more suitable models for CMBs have yet to be developed.

**Table 1:** The animal models of CMBs.

Animal models	Methods	Pathological Changes	Advantages	Disadvantages	Refs
Aged CAA-related mouse model	Tg 2576 transgenic mice	Amyloid deposits and CMBs	Mimicking cerebral amyloid angiopathy in CMBs, allowing preclinical safety evaluation of antithrombotic therapies	Taking about 15 to 24 months for CMBs development, instability in microhemorrhage size and number	[41,104-106]
	APP/PS1 mice				
	APP23-transgenic mice				
Laser-induced CMBs model	Laser pulses targeted a single cortical penetrating arteriole to produce 100 µm diameter hematoma	CMBs	Tracking the physiological changes after microhemorrhages, having specific spatial and temporal distribution, and taking less time	Skillful technique is required, invasive, hardly forming deep CMBs	[107]
Hypertension-induced cerebral microhemorrhage model	Mice were treated with Ang II and L-NAME	Hypertension and CMBs	Mimicking hypertensive vasculopathy CMBs	Taking about 15 to 24 months for CMBs development, instability in microhemorrhage size and number	[108, 110]
	IGF-1 deficiency mice with angiotensin II plus L-NAME treatment				
Hypoxia-reoxygenation-induced CMBs models	Mice were exposed to normobaric hypoxia at 8% oxygen for 48h and then kept for a further 24h at room air	CMBs	Mimicking the high-altitude hypoxic brain injury, easy to establish and taking less time	Failing to mimick the major types of CMBs’ pathogenesis	[111]
Inflammation-induced mouse model	Intraperitoneal injection of LPS	CMBs and BBB leakage	simplicity, feasibility, non-invasiveness, high success rate, and low mortality	Unable to rule out the effects of peripheral inflammation on the behavior of experimental animals	[112]
Collagenase-induced cortical CMBs murine model	Stereotaxic cortical injection of 0.8?µU collagenases	CMBs, cognitive impairments and hypometabolism	focused on cortical CMBs, sensitive to pharmacological modulation, and facilitating the assessment of cognitive and metabolic characteristics six weeks after CMBs induction	Invasive, small drug concentration range, and likely to be confounded with a larger extension of hemorrhagic lesion	[113]

CMBs, Cerebral microbleeds; CAA, Cerebral amyloid angiopathy; Ang II, Angiotensin II; L-NAME, Nω-nitro-l-arginine methyl ester hydrochloride; IGF-1, Insulin-like growth factor 1; LPS, Lipopolysaccharide; BBB, Blood-brain barrier.

## 3. The prevalence and factors influencing the clinical presentation of cerebral microbleeds

**Figure 2.**
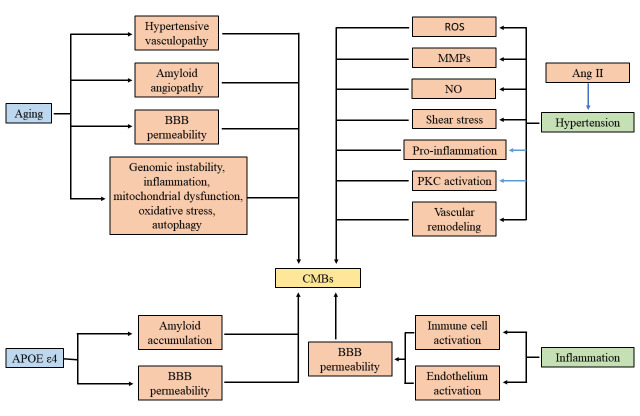
**Schematic summarizing effects of aging, hypertension, APOEε4, and inflammation for the clinical presentation of cerebral microbleeds in respect of BBB integrity.** Abbreviations: CMBs, cerebral microbleeds; BBB, blood-brain barrier; Ang II, angiotensin II; NO, nitric oxide; MMPs, matrix metalloproteinases; ROS, reactive oxygen species; PKC, protein kinase C.

Cerebral microbleeds is a cerebrovascular disease without symptoms of acute focal neurological dysfunction, and it is linked to stroke and other neurological disorders, including dementia, Parkinson’s disease, gait disturbances, and late-life depression [9, 116-118]. Meanwhile, the presence of CMBs significantly influences the progression, treatment, and prognosis of the diseases mentioned above. Accordingly, it is vital to identify CMBs risk factors at the early phase and give reasonable intervention to reduce CMBs-related hazards. There is broad recognition in numerous cohort studies that aging, hypertension, inflammation, and APOE ε4 genotype correlate with increased risk for CMBs [15, 16, 119]. In this section, we review BBB alterations and refer to common risk factors for CMBs (Fig. 2).

### 3.1 Aging

A number of studies showed that CMBs are commonly characterized as age-related cerebral microangiopathy. The prevalence of microbleeds in participants aged over 45 is 18.7%. CMBs are also found in the middle-aged population, with an increasing tendency during aging [5, 16]. The prevalence of CMBs increases with aging, from 6.5% in subjects aged 45-50 years to ~36.7% in people 80 years of age and older [16, 118]. The two types of CMBs pathogenesis, amyloid angiopathy and hypertensive vasculopathy, result in a progressive accumulative effect over time. Therefore, it is necessary to understand how aging contributes to cerebral microbleeds, especially vascular pathology. An increasing number of studies suggest that alteration of BBB permeability occurs with aging under normal and pathological conditions [119-122]. Montagne et al. found that early BBB dysfunction occurred in the hippocampus rather than other regions of the brain during normal aging via advanced dynamic contrast-enhanced magnetic resonance imaging (DCE-MRI) and post-processing analysis. It might be regarded as a cue to explore the close association between CMBs and dementia [123]. The age-related BBB leakage might contribute to the occurrence and development of CMBs. The increased prevalence of CMBs associated with aging may reflect age-related neurodegeneration and decline in cerebrovascular health. The pathophysiological roles of molecular and cellular vascular disease mechanisms, including genomic instability, inflammation, mitochondrial dysfunction, oxidative stress, and autophagy, often change with advancing age [124, 125]. Besides, cerebral microvessels are increasingly sensitive to mechanical stress with aging. However, to date, the mechanisms of aging and their underlying relationship with CMBs remain obscure and require further studies due to the deficiency of animal models suitable for this research. It might be feasible to understand how aging affects CMBs by interpreting the BBB integrity alteration under the influence of aging.

Multiple studies conducted with mice and humans demonstrated the increased BBB permeability by noting the occurrence of increased extrusion of plasma proteins (albumin, fibrinogen, and immunoglobulin G) and increased cerebrospinal fluid/plasma albumin ratios in vivo. There is also evidence proving that BBB alterations occur with aging, including a decreased number of endothelial cells, loss of TJs protein, elevated activity of GFAP^+^ astrocytes, loss of pericytes, basement membrane thickening, and reduced microvascular density and cerebral blood flow (CBF) [120, 126-128]. It is widely recognized that senescent cells accumulate in the brain with aging [129]. A study in vitro and in vivo senescent BBB models showed the association between senescent vascular cells and the dysfunction of BBB integrity. For example, aging ECs and pericytes are associated with alteration of TJs structure and dysfunction and BBB leakage in vitro, and the reduced TJ proteins coverage in vivo [130]. Recently, studies via single-cell RNA sequencing suggested that ECs in cerebral capillaries were sensitive to age-related circulatory cues at the transcriptome level and upregulate innate immunity and oxidative stress pathways in response. For example, the occurrence of increased expression of vascular cell adhesion molecule 1 (VCAM1) was counteracted by young plasma administration to reverse these changes [120, 131, 132]. In addition, senescent astrocytes induced by oxidative stress are also present with the downregulation of pro-inflammatory genes and upregulation of genes involved in neuronal generation and differentiation, and genes related to astrocytic responses, such as GFAP and the MHC class II gene [133, 134]. Changes in the astrocyte transcriptome may lead to failure in terms of response to injury under the impact of aging. Moreover, aging-induced microglia can upregulate their expression of inflammatory cytokines (IL-1α, TNF, and C1q). Aging microglia appeared to further induce A1-like reactive astrocytes, which lose their normal function and release neurotoxic factors to kill neurons and oligodendrocytes [135, 136]. In addition, the reduction in CBF and cerebral microcirculation might contribute to age-related loss of pericyte coverage. The pericyte loss has a significant role in pathological accumulations of neurotoxic macromolecules, such as hemosiderin, thrombin, and plasmin [137].

Apart from the direct impact of aging on BBB components, aging makes the brain more susceptible to inflammation-induced CMBs, related to microglial/macrophage activation [138]. Meanwhile, aging promotes the decline of circulating IGF-1 levels, which affects multiple aspects of brain health [139]. In IGF-1-deficient mice, Tarantini et al. observed a higher count of CMBs induced by hypertension. IGF-1 deficiency plays a significant role in promoting hypertension-induced MMP activation, impairing hypertrophy and structural remodeling [110].

### 3.2 Hypertension

Numerous population-based cohort studies indicate that hypertension, especially severe hypertension, is an independent risk factor for CMBs [5, 13, 15, 16]. More importantly, systolic blood pressure is regarded as a strong predictor of CMBs development. The new presence of CMBs occurs in those with much higher mean systolic blood pressure (163±20 mmHg) compared to those without (141±16 mmHg) [140]. Léon conducted a cohort study among hypertensive patients without a history of cerebrovascular disease through 24-hour ambulatory blood pressure monitoring. The results suggested that the prevalence of CMBs in hypertensive populations was approximately three times higher than that in the healthy population. The occurrence possibility of CMBs in patients diagnosed with nocturnal hypertension was approximately 5- to 6-fold higher [102]. Moreover, deep or infratentorial microbleeds are suggested to function as an independent indicator of hypertensive or arteriosclerotic microangiopathy [15].

In the pathogenesis of CMBs, hypertension-mediated vascular reactive oxygen species (ROS) production and redox-sensitive activation of MMPs play an essential part, which is related to the degradation of basement membrane components and extracellular matrix and further leads to BBB dysfunction [109]. Therefore, it is reasonable to believe that BBB dysfunction is linked to hypertension and CMBs development. It is valuable to understand how hypertension contributes to BBB breakdown. The pathogenesis of hypertension is closely related to the alteration of the renin-angiotensin-aldosterone system (RAAS), especially Ang II [141]. Nyúl-Tóth, Ádám, et al. found that there was a higher total number of CMBs in Ang II -induced hypertensive Tg2576 mice compared to normotensive Tg2576 mice [142]. As the major effector molecule of the RAAS and a potent vasoactive peptide, Ang II mediates vascular remodeling and exerts pro-inflammatory effects, further leading to disruption of the BBB integrity [143, 144]. The impairment of Ang II in BBB endothelial cells is closely involved in altering transcellular and paracellular permeability, which is associated with the activation of protein kinase C (PKC) [145]. Besides, the effects of Ang II also refer to cerebrovascular inflammation, which is partly related to oxidative stress within the SFO-PVN pathway. Chronic infusion of Ang II leads to a higher number of rolling and adherent leukocytes in mouse cerebral microvessels, and increased BBB permeability [146]. Mounting evidence suggests that the pro-inflammatory effects of central Ang II are likely to be exerted through the activation of microglia, which serves as a complementary mechanism of AngII-mediated BBB dysfunction. The activated microglia also function as a source of ROS, which might contribute to hypertension-mediated BBB breakdown [147].

CBF is essential for delivering oxygen and nutrients to cross BBB to keep normal cerebral function. It is regulated by the cerebral autoregulatory mechanism to sustain a relatively stable level by counteracting blood pressure fluctuations [148]. Nevertheless, the major impacts of constant elevated blood pressure on cerebral arteries and arterioles are hypertrophy and inward remodeling, leading to smaller external diameter, greater vascular resistance, and increased arterial stiffness [149]. Therefore, hypertension is involved in the extended range of cerebrovascular autoregulation in terms of reduced resting CBF. Autoregulatory dysfunction is associated with periventricular white matter injury [150]. Additionally, increased arterial stiffness contributes to an elevated level of pulse pressure (PP). Cerebral microvessels are more vulnerable to high pulse pressure-related mechanical stress due to their fragile structure. A clinical study for stroke confirmed that arterial stiffness was independently associated with CMBs [151]. High PP can lead to BBB dysfunction, the presence of microhemorrhages, and a reduction in microvessel density [152]. Furthermore, hypertension decreases the production of nitric oxide (NO) and increases shear stress, which are related to endothelial dysfunction and atheroma formation [153]. In terms of anatomy, the deep regions with CMBs are generally supplied by small perforating arteries, which are vulnerable to luminal narrowing, twisting, and looping, and might be aggravated by hypertension. Therefore, the distinctive hypertension-induced alterations in small arteries and arterioles increase cerebral small vascular disease susceptibility, especially CMBs.

### 3.3 APOE ε4

Several population-based studies have reported the association between the E4 variant of APOE (APOE ε4) carrier and the presence of cerebral microbleeds, especially in lobar distribution [13, 16, 154, 155]. Recently, a genome-wide association study (GWAS) of CMBs confirmed that APOE ε4 was an independent genetic risk factor for CMBs with OR value of 2.54, regardless of location [156]. Besides, the presence of APOE ε4 genotype plays a role in increasing brain amyloid load, indicating a higher risk of lobar CMBs [29]. Interestingly, several studies revealed the effect of APOE ε4 genotype on accelerating pericyte degeneration and BBB leakage in Alzheimer’s disease [157-159]. Similarly, Montagne provided the evidence by DCE-MRI that normal cognitive individuals carrying APOE4 showed increased BBB leakage in the hippocampus and medial temporal lobe compared to those without APOE4 (APOE3 homozygotes) [160]. Therefore, it is noteworthy whether the effect of APOE4 on BBB breakdown is involved in the occurrence and development of CMBs.

In the brain, APOE is mainly expressed by astrocytes and has a role in regulating lipid transport and cholesterol homeostasis as a ligand for lipoprotein receptors [161, 162]. Multiple studies suggest that APOE4 directly impacts BBB disruption and cerebral blood flow reduction compared to APOE2 and APOE3, which are other isoforms of human APOE [163, 164]. In APOE4 transgenic mice, the increased BBB permeability is detectable, including leakage of multiple blood-derived neurotoxic proteins, diminished pericyte coverage and microvascular length, enzymatic degradation of basement membrane proteins and TJs mediated by MMP-9, and reduction of regional CBF [164, 165]. APOE4 proteins participate in the process of BBB breakdown primarily through increasing cyclophilin A (CypA) expression in brain capillary pericytes and regulating the pro-inflammatory CypA-NF-κB-MMP9 pathway. MMP-9 is related to the degradation of the basement membranes and TJs, subsequently causing the BBB leakage [165, 166]. Moreover, APOE4 proteins contribute to cerebral Aβ accumulation in the cerebral parenchyma and microvessels through preventing Aβ clearance in the form of APOE-bound Aβ, which was confirmed as an important part of CAA [167-170]. There appears to be greater affinity between APOE4 and VLDL receptor (VLDLR) than LDL receptor-related protein 1 (LRP1), which mainly mediates Aβ clearance across the BBB. There is a much lower rate of clearance of Aβ-APOE4 complexes mediated by VLDLR than LRP1-mediated Aβ binding, endocytosis, and transcytosis, which leads to Aβ deposition in cerebral microvessels [171-173]. In vitro and vivo studies have shown that neuroinflammation and ROS are involved in TJ disruption and microglia recruitment in capillary CAA [174, 175]. Furthermore, the accumulation of Aβ in CAA contributes to the increased BBB permeability by inducing MMP-9 activity and reducing the expression of TJs [173, 176, 177].

### 3.4 Inflammation

Inflammation exerts the role as an essential part of CMBs pathogenesis and one of CMBs’ most significant risk factors. A pilot cross-sectional study showed an association between the presence of CMBs and infection with multiple pathogens, such as herpes simplex virus (HSV)-1 and HSV-2 [178]. Histopathological evidence demonstrated that hemosiderin deposits at the lesion site of CMBs are often surrounded by macrophages, a type of immune cells that further initiate inflammatory responses. The presence of CMBs is closely associated with an elevated level of circulating inflammatory biomarkers, such as high-sensitivity C-reactive protein (hsCRP), interleukin (IL)-6, IL-18, tumor necrosis factor receptor 2 (TNFR2), and myeloperoxidase [179-181]. Both vascular inflammation and systemic inflammation are reported to contribute to the occurrence and development of CMBs. Vascular inflammation seems to be related to hypertensive arteriopathy and BBB disruption, while systemic inflammation is likely involved in CAA-related microvascular pathological variation in cortical regions [181]. The inflammation-induced CMBs animal model in which the animals were treated with LPS confirmed the importance of systemic inflammation in the pathogenesis of CMBs, such as brain endothelium activation, BBB disruption, and neuroinflammation [112]. In addition to its role as one of the risk factors for developing CMBs, inflammation might also play an essential role in mediating further impairment of neuronal function after a microhemorrhage. Ahn et al. adopted in vivo 2-photon excited fluorescence microscopy to follow the inflammatory response in real-time after laser-induced cortical CMBs occurred. The study indicated that CMBs were involved in the inflammatory response, which lasted for more than a week, including activation of microglia and astrocytes, and the invasion of blood-borne CX3CR1^+^ and CCR2^+^ macrophages [182].

Despite the fact that the exact molecular mechanisms of inflammation with respect to CMBs have not yet been well clarified, loss of BBB integrity associated with inflammatory responses might potentially participate in CMBs development. Inflammation may thus lead to BBB dysfunction that gives rise to the development and progression of CMBs. The contribution of inflammatory mediators to BBB breakdown can be described by the following three aspects. First, circulating inflammation leads to microglia recruitment to cerebral vessels and elevated expression levels of paracellular TJs, which are initially conducive to BBB integrity. When persistent inflammation occurs, the microglia increase the expression of the phagocytic marker CD68 and phagocytosis of astrocyte end-feet [183]. Second, inflammation has a role in modulating TJ expression, increasing MMP-mediated enzymatic degradation of TJs, and forming vesicular transendothelial channels, which leads to reduced material transport via paracellular pathways and transcytotic vesicular pathways [184, 185]. Third, loss of endothelial integrity is commonly present in cerebral inflammation, and includes apoptotic cell death, impairment of transporter activity, and damaged organelles [185, 186]. In addition, endothelial cells are sensitive to inflammation. They increase the expression of chemokines and cell adhesion molecules to facilitate the recruitment and migration of circulating immune cells to the brain [187]. All of the mechanisms mentioned above are related to the BBB breakdown due to the interference of inflammation mediators.

## 4. The vulnerability of the BBB to cerebral microbleeds

An increasing number of clinical studies have demonstrated the association between the presence of CMBs and the BBB breakdown through various biomarkers existing in CSF and serum, including increased serum VEGF and fibrin levels, reduced MMP-9 levels in CSF, elevated levels of contrast agent leakage, and altered CSF/serum albumin ratio [48, 188]. Meanwhile, numerous animal studies have verified the correlation between CMBs development and BBB dysfunction via contrast agent leakage, plasma protein extravasation, gelatinase expression alteration, and IgG deposition [189]. Due to the barrier effect of the BBB, the increased permeability of the BBB plays an essential role in reducing cerebral blood flow and impairing hemodynamic responses. Particularly, neuroinflammation significantly contributes to the breakdown of the BBB, which is recognized as an essential activation event during the early phase of neurological system diseases, such as stroke, Alzheimer’s disease, and multiple sclerosis [185, 190].

**Figure 3.**
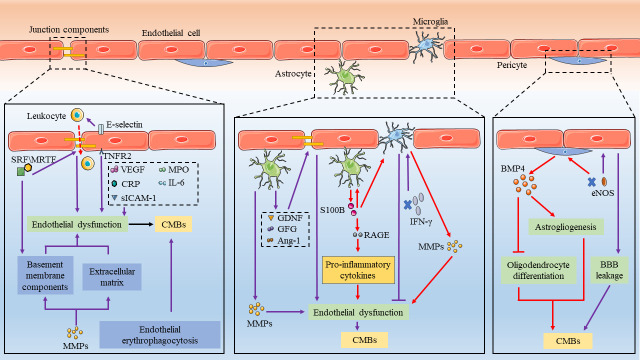
**Schematic representation of blood-brain barrier (BBB) alteration in cerebral microbleeds (CMBs).** Cell-cell interactions in the neurovascular unit indicate the breakdown of BBB and promote CMBs development. VEGF, CRP, sICAM, MPO, IL-6, and E-selectin contribute to endothelial dysfunction. Serum Response Factor (SRF) and its MRTF cofactors play a vital role in cerebral microvascular integrity through regulating EC junction components and basement membrane proteins. Matrix metalloproteinases (MMPs) derived from microglia and astrocytes are associated with the degradation of TJs and ECM, exacerbating the injury of the vascular wall. S100B derived from astrocytes is able to promote the release of oxidative stress mediators and pro-inflammatory cytokines, resulting in further BBB breakdown and the development of CMBs. At the same time, the cytokines derived from microglia and astrocytes play a role in endothelial dysfunction, such as glia-derived neurotrophic factor (GDNF), fibroblast growth factor (FGF), angiopoietin 1 (Ang-1), and IFN-γ. Recent studies also suggest a role of pericytes in the development of CMBs through the bone morphogenetic protein 4 (BMP4) pathway, which is related to astrogliogenesis and inhibits oligodendrocyte differentiation.

Under the impact of multiple risk factors, the structure and function of the BBB are damaged due to the alteration of endothelial cells, pericytes, astrocytes, and the basement membrane, which causes small vessels to rupture in the context of CAA or hypertension. The increased BBB permeability leads to erythrocyte exudation from small cerebral vessels, which is the primary characteristic of CMBs. Additionally, dysfunction of the BBB enables neurotoxic blood-derived components, blood cells, and pathogens to enter the brain tissues, which causes deterioration of the cerebral environment, further brain tissue injury, and aggravation of CMBs development. However, the cellular and molecular mechanisms underlying BBB dysfunction associated with CMBs development remain to be identified. Here, we provide an overview of existing studies on pathological structural alterations of BBB components and their roles in the occurrence and development of CMBs (Fig. 3).

### 4.1 Endothelial dysfunction

Compared with endothelial cells in the peripheral tissue, the continuous endothelial monolayer within the BBB lacks fenestrations and has the capability to strictly regulate the efflux and influx of ions, toxins, blood cells, nutrition, and pathogens by its unique permeability properties. The adjacent ECs are linked by the junction complex predominately comprising TJs and adherens junctions at the ultrastructural level, which contribute to limiting the diffusion of most hydrophilic molecules from plasma to the CNS through the paracellular pathway and the subsequent creation of high transendothelial electrical resistance (1500-2000 omega•cm^2^) of the BBB [20, 191-193]. In addition, the maintenance of normal BBB physiological function is inseparable from the unique transport systems, including influx and efflux transporters, limited transcytosis rate, and low level of leukocyte adhesion molecules [17, 190].

Endothelial cells are essential for maintaining vascular homeostasis due to their capability of perceiving alterations in the hemodynamic forces and blood-derived factors. They can give a timely response through releasing substances involved in different pathways of endothelial functions, including regulating vascular tension, participating in inflammatory responses, regulating fibrinolysis and coagulation pathways, and playing a role in vessel formation, repair, and remodeling [194-196]. Considering the extensive effects of ECs, it is no wonder that endothelial dysfunction takes center stage in the pathogenesis of cerebrovascular diseases, CMBs included. Weinl found that *Srf^iECKO^* mice, in which serum response factor (SRF) is depleted, are prone to develop macro- and microhemorrhages. SRF and its MRTF cofactors play a vital role in cerebral microvascular integrity by regulating EC junction components, such as claudins, ZO adapter proteins, actin, and basement membrane proteins [197]. Alomar et al. observed increased levels of BSA-FITC leakage from arterioles with diameters of 20-50 μm in type 1 diabetic rats, which resembled CMBs. In contrast, BSA-FITC transcytosis was blunted by reduced methylglyoxal under the regulation of methylglyoxal-degrading enzyme glyoxalase-I (Glo-I) in smooth muscle cells in cerebral arterioles [198]. However, there are few directly relevant studies on transcellular leakage during the development of CMBs, which requires further exploration.

Clinical studies revealed exclusive associations between CMBs and endothelial dysfunction markers, E-selectin, and vascular endothelial growth factor (VEGF), rather than other cerebral small vessel disease (cSVD) markers [179, 181, 199]. E-selectin is a glycoprotein adhesion molecule that is specifically expressed in activated ECs. It promotes the migration of leukocytes into the arterial wall and mediates inflammatory cascades [200]. There is a significant correlation between serum VEGF level and the number of CMBs in patients with Alzheimer’s disease [201], while similar results were also found in patients with acute ischemic stroke [202]. As an essential effector in regulating microvascular density and permeability, VEGF can impair the BBB integrity and further disrupt CNS homeostasis [203]. VEGF-A inhibits the expression of claudin-5 and occludin in brain microvessel endothelial cell cultures in vitro and in the CNS in vivo, which causes increased paracellular permeability [204]. The levels of serum soluble intercellular adhesion molecule 1 (sICAM-1) significantly correlate with the presence of CMBs and the hemorrhagic transformation risk, and sICAM-1 plays a role in endothelial dysfunction and inflammatory responses [205]. Higher levels of circulating inflammatory biomarkers, such as TNFR2, myeloperoxidase, CRP, and IL-6, have been previously reported in patients with CMBs [179, 206]. Because the multiple endothelial circulating biomarkers cannot exactly reflect the alteration of the brain endothelium, it is essential to find specific biomarkers corresponding to cerebral endothelial dysfunction. Moreover, the issue of causality between endothelial dysfunction and the presence of CMBs remains to be further explored.

Apart from inflammation responses and TJ breakdown, endothelial erythrophagocytosis might also contribute to the occurrence of CMBs. Multiple studies have shown endothelial cells are involved in the phagocytosis of aged or apoptotic erythrocytes upon phosphatidylserine exposure [207]. Chang et al. studied the relationship between the phagocytosis of red cells and CMBs based on a cerebral microbleeds model in vitro. There was more significant cerebral endothelial phagocytosis of erythrocytes exposed to oxidative stress compared to the control group. The promoted endothelial erythrophagocytosis was mediated by the passage of hemoglobin across brain endothelial cells without any alteration in monolayer integrity [208]. Additionally, a portion of endothelial cells showed signs of apoptosis after the phagocytosis of red cells, which might affect intracellular processes or the release of oxidized free heme [209]. However, the detection of CMBs mainly depends on hemosiderin’s paramagnetic properties, and thus, the presence of CMBs can be recognized by any passage of hemosiderin or erythrocytes through endothelial cells. Therefore, it is worth considering the potent mechanisms of CMBs without microvessel rupture.

### 4.2 Cross-talk among the BBB cellular components in CMBs

Pericytes are located adjacent to tight junctions and gaps between endothelial cells, and they function as a ‘gatekeeper’ in the BBB. Pericytes possess contractile properties that directly regulate the CBF through the constriction of capillaries, which is initiated by pericytes under the stimulation of ATP and noradrenaline [210]. Pericytes can regulate gene expression in endothelial cells and contribute to BBB permeability, for example, by upregulation of TJ proteins and upregulation of Mfsd2a to suppress endothelial transcytosis [17]. An increasing number of studies have recently identified the multipotential stemness of pericytes, and especially their capacity to differentiate into neural and vascular lineage cells under ischemia/hypoxia. Furthermore, pericytes are considered as an underlying resource that can be used for restoration of the BBB after brain damage [211]. Pathological analysis of post-mortem brain specimens demonstrated pericyte involvement in 2 out 22 cases with CMBs that were immediately adjacent to endothelial TJs [212]. The activation of the CypA-MMP-9 pathway in pericytes promotes the degradation of TJ proteins and basement membrane proteins. Recently, in a spontaneous cSVD animal model with partial eNOS deficiency, there was increased BBB breakdown in aged mice, and pericyte-derived bone morphogenetic protein 4 (BMP4) in eNOS-deficient mice was elevated. BMP4 accelerates astrogliogenesis and inhibits oligodendrocyte differentiation, which further leads to microbleeds, white matter pathology, and neurodegeneration [213].

Astrocytes play a vital role in narrowing the gap between capillaries and neurons due to their contribution to synapse formation, BBB formation and maintenance, and CNS homeostasis [214]. Astrocytes contribute to BBB permeability mainly through releasing a series of cytokines, such as glia-derived neurotrophic factor (GDNF), fibroblast growth factor (FGF), and growth factors such as Ang-1, that affect TJ expression and EC activity [215-217]. Microglia act as continuous immune surveillant cells in the brain. They can monitor the CNS environment for pathogens and be activated by stimuli to protect the brain. Activated microglia have the ability to engulf microorganisms, transform to an M1/M2 phenotype, and release a series of inflammatory mediators [218]. Furthermore, activated microglia can trigger reactive astrocytes and amplify neuroinflammation, while astrocytes are able to sensitively identify minor changes in neurons and vessels, and deliver signals to microglia [219]. In the laser-induced microhemorrhage model, microglia migrated to the lesion during the early stages, while the activation of astrocytes was delayed for several days [182]. In the LPS-induced animal model, the CMBs burden was significantly associated with total Iba1- and GFAP-positive immunoreactive areas and ICAM-1-positive areas, which was related to the activation of microglia, astrocytes, and endothelial cells [112]. Furthermore, in diabetic mice, there were reduced microglial polarization and accumulation near the microvascular injury, lesions, concomitant with increased BBB leakage. The inhibition of IFN-γ promoted microglial function impairment and reduced BBB dysfunction [220].

Apart from animal studies, a clinical study confirmed the role of astrocytes in the presence and number of deep CMBs, with reduced soluble receptors for advanced glycation end products (sRAGE) levels and increased S100B levels [221]. S100B is a member of S100 protein family of Ca^2+^-binding proteins, mainly expressed in astrocytes [222]. S100B plays a neurotrophic role in facilitating neuronal proliferation, oligodendrocyte differentiation, and astrocyte and microglia migration, with concentrations in the nanomolar range. In contrast, micromolar concentrations of S100B can induce pro-inflammatory effects through the activation of RAGE. The high concentrations of S100B derived from astrocytes lead to astrocyte and microglial activation and neuronal death. S100B can also promote the release of oxidative stress mediators and pro-inflammatory cytokines, resulting in further BBB breakdown and the development of CMBs [223]. More importantly, multiple studies reported the correlation between CMBs development and MMPs, especially MMP-2 and MMP-9 [61, 224, 225]. MMPs are a family of zinc-dependent endoproteinases that can be partially secreted by astrocytes and microglia [224]. MMPs activation is commonly observed in CMBs animal models under the inducement of oxidative stress. Activated MMPs can damage the basal lamina and degrade TJs and the extracellular matrix (ECM), exacerbating vascular wall injury.

## 5. Potential pharmacological approaches to target the BBB as therapy for CMBs

An increasing number of studies have noted the significance of CMBs in the prevention and treatment of stroke, which includes antihypertensive treatment, antiplatelet therapy, thrombolysis, anticoagulant therapy, and statin therapy. The presence of CMBs is closely related to subsequent ICH and recurrent ischemic stroke in patients with recent ischemic stroke or TIA under antithrombotic treatment [6]. Additionally, CMBs might participate in the progression of dementia, which seems to be delayed with early intervention to CMBs. Therefore, it is essential to devise potential pharmacological interventions to inhibit or reduce CMBs development. In particular, BBB is a promising therapeutic target for CMBs.

A growing number of studies have indicated that vascular ROS production and redox-sensitive activation of MMPs are essential elements of CMBs development, which is interrelated with damage to the basal lamina and degradation of TJs and ECM. Thus, antioxidants and MMPs inhibitors are a likely option for the treatment of CMBs. The two different anti-ROS interventions, apocynin and tempol, played a significant role in improving cerebrovascular function via attenuating CAA formation and CAA-induced vasomotor dysfunction in aged Tg2576 mice. More importantly, the NADPH oxidase inhibitor, apocynin, reduced CAA-related CMBs [68]. Furthermore, resveratrol treatment has protective effects in the development of hypertension-induced CMBs in aged mice. Resveratrol reduces vascular ROS production via the downregulation of the NADPH oxidase subunits and disruption of MMPs activation [109]. A study in aged Tg2576 mice indicated that chronic minocycline treatment inhibited MMP-2 and -9 activity, and attenuated gliosis, gelatinase activity, and inflammation, which further resulted in reduced hemorrhage frequency [226].

A randomized controlled trial suggested that cilostazol, a type III phosphodiesterase (PDE3) inhibitor, significantly reduced the incidence of cerebral hemorrhage compared with aspirin in patients with multiple CMBs [227]. The post hoc analysis of this trial showed that there was lower ICH risk with the use of cilostazol, and it tended to reduce the composite of major vascular events compared to aspirin in the CMBs subgroup [228]. Studies in vivo demonstrated that cilostazol appeared to provide protective effects to BBB properties. Cilostazol decreased paracellular and transcellular permeability, promoted the expression of claudin-5, and regulated actin cytoskeleton rearrangement [229]. However, Sumbria et al. found that pharmacological inhibition by cilostazol failed to modulate CMBs development in both LPS-induced models and CAA-related models. The failure appeared to be consistent with no reduction in endothelial, astrocyte, or microglial activation, or BBB injury [230].

Histologically, the vascular rupture in CMBs leads to the leakage of erythrocytes from small cerebral vessels, and the formation of hemosiderin deposits near the lesions [4]. The iron derived from heme degradation is closely related to the secondary brain damage mediated by excessive production of free radicals [40]. Iron dyshomeostasis induces BBB dysfunction and microglial activation, and further leads to dendrictic degeneration. Deferoxamine is approved for the treatment of chronic iron overload and acute iron intoxication. CMBs induced by two-photon lasing were ameliorated by deferoxamine treatment, which was associated with a reduction in iron deposits and reactive microglia [27]. Therefore, deferoxamine has significant potential to be used as a treatment for CMBs.

The pharmacological approaches targeting the BBB mentioned above provide potential novel interventional strategies for CMBs. However, most of these interventions are still at the preclinical phase, and their treatment effects might vary unpredictably when further applied to large numbers of patients. Therefore, further investigation is needed to translate these laboratory findings to the clinic. CMBs are related to multiple alterations of the BBB components in various pathways, and single-target drugs appear to have limited impact. Thus, the application of the combination of vasoprotective drugs and other interventions may be more effective. Moreover, the relationship between CMBs and the side effects of common antithrombotic therapies remains controversial. Further preclinical studies are required to improve our understanding of the diagnostic and therapeutic significance of CMBs when they occur under multiple pathological conditions.

### Conclusion

Although there have been numerous studies on CMBs, the existing research has concentrated on the clinical significance of CMBs. The exact mechanisms of vascular pathology and BBB alterations are still far from clear. There also exist a number of controversies on prevention and management, including the use of therapies with antiplatelet, anticoagulant, lipid-lowing, and thrombolytic actions. Furthermore, greater emphasis on understanding CMBs-related clinical consequences, especially stroke and dementia, is necessary, as well as additional interventional strategies to ameliorate or prevent CMBs.

Considering their clinical significance, it is necessary to place greater emphasis on studying CMBs and finding potential preventive and treatment strategies. BBB dysfunction is considered to initiate the occurrence of CMBs. However, the main difficulty in investigating CMBs is the deficiency of ideal animal models, and thus, it is difficult to conduct additional exploration to elucidate the advanced mechanisms of BBB that contribute to CMBs. Additional definitive studies are needed to understand how CMBs contribute to the different outcomes of ischemic stroke under antithrombotic therapy.

In conclusion, despite many details that still require study, considerable evidence suggests that BBB dysfunction appears to play a significant role in the development and progression of CMBs. Risk factors for CMBs can exacerbate BBB breakdown through the vulnerability of the BBB to anatomical and functional changes. To reduce the burden of CMBs, it is necessary to increase awareness of BBB alterations and perform additional research to increase our knowledge regarding their relationship with CMBs.
